# Downregulation of PD-L1 expression by Wnt pathway inhibition to enhance PD-1 blockade efficacy in hepatocellular carcinoma

**DOI:** 10.1186/s13062-025-00645-8

**Published:** 2025-04-10

**Authors:** Yu-Yun Shao, Han-Yu Wang, Hung-Wei Hsu, Rita Robin Wo, Ann-Lii Cheng, Chih-Hung Hsu

**Affiliations:** 1 Graduate Institute of Oncology, National Taiwan University College of Medicine, 1, Sec. 1, Ren’ai Rd, Taipei City, 10051 Taiwan; 2 Department of Internal Medicine, National Taiwan University College of Medicine, 1, Sec. 1, Ren’ai Rd, Taipei City, Taiwan; 3 Department of Oncology, National Taiwan University Hospital, 7, Chun-Shan S. Rd, Taipei, Taiwan; 4 Department of Medical Oncology, National Taiwan University Cancer Center, 57, Ln. 155, Sec. 3, Keelung Rd, Taipei City, Taiwan

**Keywords:** Beta-catenin, Hepatocellular carcinoma, Immunotherapy, Programmed death-ligand 1, Tankyrase, Wnt pathway

## Abstract

**Background:**

Immunotherapy targeting the programmed death-ligand 1 (PD-L1) pathway is a standard treatment for advanced hepatocellular carcinoma (HCC). The Wnt signaling pathway, often upregulated in HCC, contributes to an immunosuppressive tumor microenvironment. This study investigated the impact of Wnt pathway inhibition on PD-L1 expression in HCC and evaluated the potential therapeutic benefit of combining Wnt pathway inhibition with PD-L1 blockade.

**Methods:**

The effects of Wnt pathway inhibitors XAV939 and IWR-1 on PD-L1 expression were examined in human HCC cell lines HuH7 and Hep3B. Beta-catenin overexpression and knockdown experiments confirmed these findings. For in vivo efficacy, the BNL 1ME A.7R.1 mouse HCC cell line was orthotopically implanted in mice, which were subsequently treated with XAV939, anti-PD-L1 antibodies, or both.

**Results:**

Wnt pathway inhibitors XAV939 and IWR-1 significantly reduced PD-L1 protein expression in a dose-dependent manner in HuH7 and Hep3B cells, without affecting mRNA levels. *CTNNB1* knockdown produced similar results, and beta-catenin overexpression reversed the effects of Wnt pathway inhibitors on PD-L1 expression. Wnt pathway inhibition did not promote PD-L1 protein degradation but instead increased the level of unphosphorylated 4EBP1, which could prevent the translation function of eIF-4E. In vivo, mice treated with a combination of XAV939 and an anti-PD-L1 antibody had significantly smaller tumors compared to those treated with either agent alone. The combination treatment also enhanced multiple immune-related pathways in harvested tumors.

**Conclusion:**

Inhibition of the Wnt pathway reduced PD-L1 expression in HCC cells and enhanced the efficacy of PD-L1 blockade, supporting its potential as HCC treatment.

**Supplementary Information:**

The online version contains supplementary material available at 10.1186/s13062-025-00645-8.

## Introduction

Despite significant advances in oncology, hepatocellular carcinoma (HCC) remains a formidable challenge, especially in advanced stages. Recent developments in immunotherapy, including the use of checkpoint inhibitors such as anti-programmed cell death protein-1 (PD-1) and anti-programmed death-ligand 1 (PD-L1) antibodies, have introduced new treatment avenues. Combination therapies involving PD-1 blockade are now the standard first-line treatment for advanced HCC [1–6]. However, not all patients benefit from these treatments, highlighting the variability in treatment outcomes and the development of resistance by some initially responsive tumors. The prognosis for patients after the failure of first-line PD-1 blockade therapy remains poor [7], underscoring the urgent need for novel therapies that can enhance the efficacy of PD-1 inhibitors or counteract resistance.

The Wnt pathway is frequently upregulated in HCC, contributing to the maintenance of tumor-initiating cells, drug resistance, tumor progression, and metastasis [8–10]. Inhibition of this pathway has been shown to improve the efficacy of targeted therapies like sorafenib in HCC [11]. Furthermore, activation of the Wnt pathway can lead to an immunosuppressive tumor microenvironment, potentially reducing the effectiveness of immune checkpoint inhibitors [12, 13]. Poorer survival outcomes have been associated with activating alterations in the Wnt pathway signaling among patients treated with immune checkpoint inhibitors [14]. 

Wnt pathway signaling has been shown to modulate the expression of PD-L1 in various cancers [15–17], enabling cancer cells to evade antitumor immunity [18, 19]. Given the multiple Wnt pathway inhibitors currently under development, this study aims to explore the impact of Wnt pathway inhibition on PD-L1 expression in HCC cells and the potential of combining Wnt pathway inhibitors with PD-1 blockade as a treatment strategy for HCC.

## Materials and methods

### Cell lines and compounds

The human HCC cell lines HuH7 (RRID: CVCL_0336) and Hep3B (RRID: CVCL_0326) were purchased from the Japanese Collection of Research Bioresources Cell Bank, and the mouse HCC cell line BNL 1ME A.7R.1 (BNL) (RRID: CVCL_6371) was purchased from American Type Culture Collection. All cell lines were maintained in Dulbecco Modified Eagle Medium with 10% fetal bovine serum, L-glutamine (2 mM), penicillin (100 units/mL), streptomycin (100 µg/mL), and amphotericin B (25 ng/mL) at 37 °C in a humidified incubator with 5% CO_2_. All human cell lines have been authenticated using STR profiling within the last three years. All experiments were performed on mycoplasma-free cells.

For bioluminescence imaging, we established BNL-Luc cells by transfected BNL cells with pLAS3w.FLuc.Ppuro lentivirus (National RNAi Core Facility of Academia Sinica, Taipei, Taiwan) in 1 ng/ml polybrene medium for 24 h. Cells were then selected using 10 µg/ml puromycin and maintained in the aforementioned condition containing 10 µg/ml puromycin.

Both human and mouse interferon-γ (IFN-γ) were purchased from PeproTech (Rehovot, Israel). XAV939, IWR-1, and cycloheximide (CHX) were purchased from Selleckchem (Houston, TX, USA).

### Western blot analysis

All Western blot analyses were conducted in accordance with the standard protocol and the suggestions of the antibody manufacturers. The following antibodies were used: β-catenin (1:1000, #9587, Cell Signaling Technology, RRID: AB_10695312), active β-catenin (1:1000, #8814, Cell Signaling, RRID: AB_11127203), interferon regulatory factor-1 (IRF-1) (1:1000, sc-497, Santa Cruz, RRID: AB_631838), PD-L1 (E1L3N) (1:1000, #13684, Cell Signaling, RRID: AB_2687655), Flag (1:2000, #2368, Cell Signaling, RRID: AB_2217020), GAPDH (1:10000, #2118, Cell Signaling, RRID: AB_561053), phospho-4E-BP1 (1:2000, #9451, Cell Signaling, RRID: AB_330947), non-phospho-4E-BP1 (1:2000, #9423, Cell Signaling, RRID: AB_491012).

### Flow cytometry

After the HCC cells were treated with Wnt pathway inhibitors and stimulated by IFN-γ, they were collected and stained with PE Mouse Anti-Human CD274 antibody (BD Biosciences, RRID: AB_647198) or PE Mouse IgG1, κ Isotype Control (BD, RRID: AB_396091) for 30 min. Detection was then carried out using a BD FACSCalibur flow cytometer.

### Quantitative reverse transcription PCR analysis

Total RNA was extracted using Direct-zol™ RNA Miniprep (Zymo Research, Irvine, CA, USA) according to the manufacturer’s instructions. cDNA synthesis was performed with the mixture of random primers using ReverTra Ace^®^ (PURIGO Biotechnology, Taipei, Taiwan). We performed quantitative reverse transcription PCR (RT-qPCR) using qPCRBIO SyGreen Blue Mix Hi-ROX (PCR Biosystems, London, UK) in accordance with the standard protocol. Glyceraldehyde-3-phosphate dehydrogenase (GAPDH) was used as the internal control in the experiments involving the human and mouse cells, respectively. The primer sequences were 5′- TGCTGAACGCATTTACTGTC (forward) and 5′- ATCTGAAGTGCAGCATTTCC (reverse) for human CD274; 5′- TGATGACATCAAGAAGGTGGTGAAG (forward) and 5′- TCCTTGGAGGCCATGTAGGCCAT (reverse) for human GAPDH.

### Reporter assay

Following the manufacturer’s recommendations, seeded cells were transfected with the TCF/LEF reporter plasmid (#CCS-018 L, QIAGEN, Hilden, Germany) using Maetrofectin (OmicsBio). After 24 h of transfection, the cells were treated with the Wnt pathway inhibitors and stimulated by IFN-γ as previously described. Luciferase activity was detected 72 h post-transfection using the Dual-Glo^®^ Luciferase Assay System (Promega, Madison, WI, USA).

### *CTNNB1* knockdown and overexpression

We purchased shRNAs from the RNA Technology Platform and Gene Manipulation Core Facility in Taiwan. HCC cells were seeded and then transfected with lentivirus particles in a medium containing 1 ng/ml polybrene for 24 h. Subsequently, stable cells were selected using 1 µg/ml puromycin. Target sequences was 5′- TCTAACCTCACTTGCAATAAT for sh-CTNNB1_#921 (TRCN0000314921) and 5′- ATCTGTCTGCTCTAGTAATAA for sh-CTNNB1_#990.

Vectors overexpressing *CTNNB1* were purchased from OriGene (RC208947, Rockville, MD, USA). After 1.5 × 10^5^ HuH7 cells had been seeded in 6-well plates, we performed transfection with Maestrofectin transfection reagent (OmicsBio, New Taipei City, Taiwan) and allowed the cells to incubate for 48 h.

### Immunohistochemical staining

We retrieved HCC tissue sample from patients who underwent curative surgery for HCC at National Taiwan University Hospital in 2015. We deparaffinized and hydrated archival formalin-fixed paraffin-embedded tissue sections with 4-µm thickness and retrieved the antigen using a citric acid buffer (pH 6.0) or Tris-EDTA buffer (pH9.0), autoclaved at 125°C for 10 minutes. The tissue slides were incubated in a dual endogenous enzyme block (#S2003, Dako) for 10 min at room temperature to stop endogenous peroxidase activity and then conjugated with primary antibodies, either anti-PD-L1 (E1L3N) (1:350, #13684, Cell Signaling) or anti-β-catenin (1:500, #6101454, BD, RRID: AB_397555) and secondary antibodies using Dako REAL EnVision-HRP Rabbit-Mouse (#K5007, Dako). We then colorized the tissue slides diluted 3, 3’-diaminobenzidine tetrachloride solution (#K5007, Dako) and counterstained them with hematoxylin. We then examined whether beta-catenin was expressed in the nucleus and evaluated PD-L1 by counting the percentages of cells with positive staining. This study was approved by the Research Ethics Committee of National Taiwan University Hospital for the use of HCC tissue samples of patients.

### Orthotopic tumor studies

BALB/c (RRID: MGI: 5568760) male mice 5 weeks of age were purchased from the National Laboratory Animal Center in Taiwan. After treated with ensuring sufficient anesthesia, a minor incision was made on each mouse’s upper abdomen to reveal the liver. Subsequently, 3 × 10^5^ BNL-Luc cells were injected into the liver’s subcapsular region, and the incision was sealed. A week later, mice were randomized to receive vehicle (4% DMSO in corn oil and IgG control, RRID: AB_1107780), 10 mg/kg XAV939, 5 mg/kg anti-PD-L1 antibody (10 F.9G2, Bio X Cell, RRID: AB_10949073), or a combination of XAV939 and anti-PD-L1 antibody. Treatment was given as intraperitoneal injections twice weekly for two weeks. The mice were then sacrificed. However, for survival analysis, euthanasia was only carried out if the mice satisfied the conditions for animal euthanasia. All animals in this study received human care and that study protocols complied with the institution’s guidelines and the Animal Research: Reporting of In Vivo Experiments (ARRIVE) guidelines. The study was approved by National Taiwan University College of Medicine and College of Public Health Institutional Animal Care and Use Committee, approval number 20,210,286.

### Bioluminescence imaging and analysis

For monitoring tumor growth in the orthotopic tumor model, we gave intraperitoneal injection of D-luciferin (150 mg/kg) to the BALB/c mice, 10 min prior to bioluminescence imaging using IVIS Spectrum system. The total photon flux (photons per second) was quantified from a predetermined region of interest using the Living Image software (Xenogen, RRID: SCR_020901) for analysis.

### Immune-related gene expression profiling

The nCounter^®^ analysis system from NanoString Technologies was employed to analyze gene expression in mouse tumor tissues, utilizing the PanCancer Immune Profiling Panel to measure more than 700 genes. Target gene raw counts were normalized against the geometric mean of 16 housekeeping genes (Abcf1, Edc3, Eef1g, G6pdx, Gusb, Hdac3, Nubp1, Oaz1, Polr1b, Polr2a, Ppia, Rpl19, Sap130, Sf3a3, Tbp, Tubb5) and controls, with a threshold of 20 for background normalization, processed by nSolver Analysis Software v4.0 (RRID: SCR_003420) and R-package (NanoString Technologies Inc., Seattle, DC, USA). Only genes with a significant difference and at least 2-fold differential expression between groups were analyzed. The heatmap of pathway analysis was plotted using Heatmapper [20]. 

### Statistical analysis

All statistical analyses were performed using SAS (9.4, SAS Institute, Cary, NC, USA). A 2-sided *p* value of < 0.05 was considered statistically significant. For continuous variables, such as expression levels, an independent *t* test was used to compare the results between the groups. Mouse survival was calculated from the day of treatment initiation and estimated using the Kaplan-Meier method. The log-rank test for trend was used to compare group survival.

## Results

### Wnt pathway inhibition reduced PD-L1 protein expression, but not mRNA expression

**Fig. 1:**
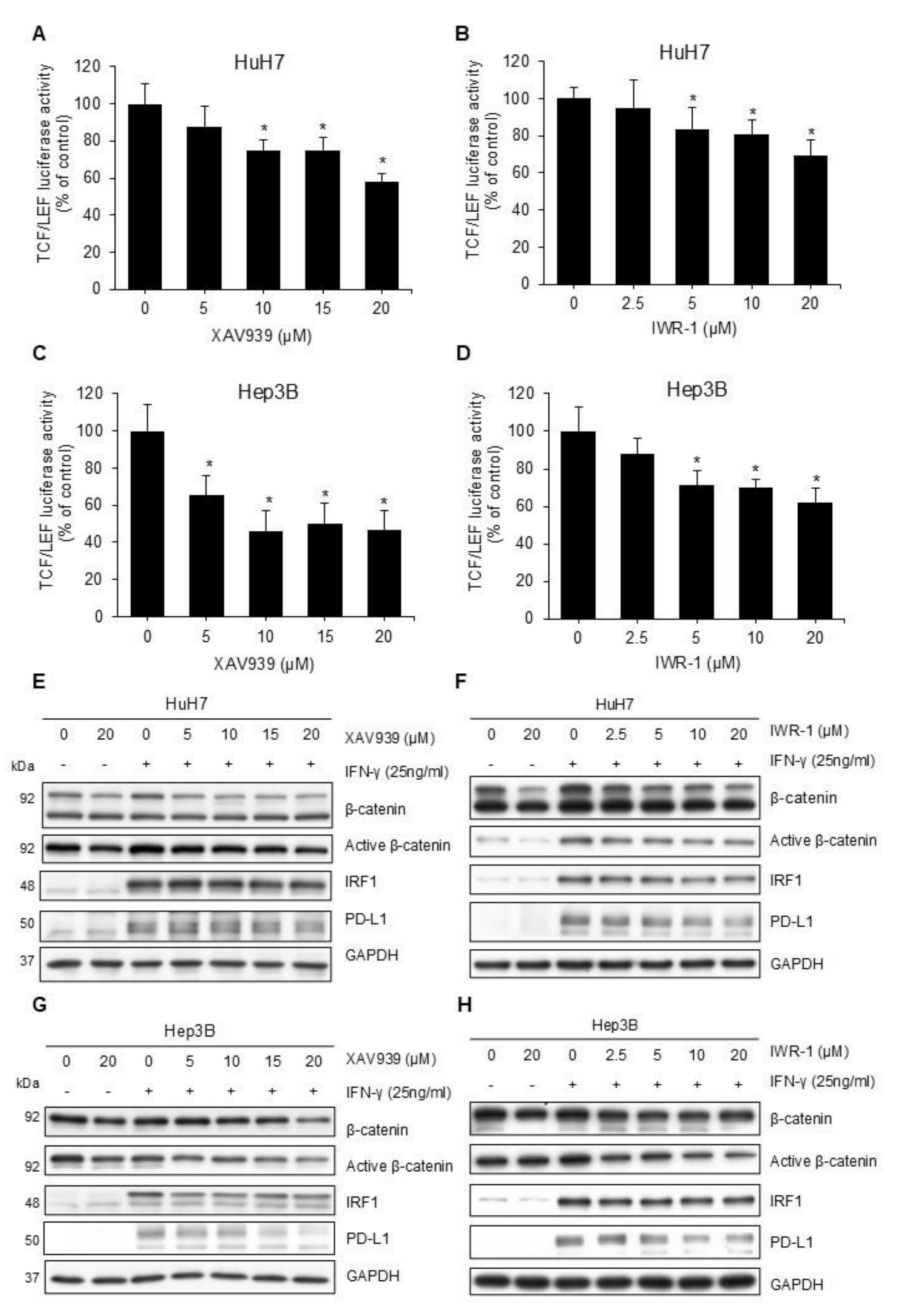
**(A-D)** Reporter assays demonstrating Wnt pathway activity. Approximately 16 h after seeding, HuH7 (**A**-**B**) and Hep3B (**C**-**D**) cells were transfected with the TCF/LEF reporter plasmid. After 24 h, the cells were treated with Wnt pathway inhibitors XAV939 (**A** and **C**) or IWR-1 (**B** and **D**) at the indicated concentrations. Luciferase activity was detected 48 h after Wnt pathway inhibitor treatment and presented as fold changes relative to cells treated with the vehicle. (^*^p < 0.05). **(E-H)** Western blotting demonstrating PD-L1 expression in HCC cells. Approximately 16 h after seeding, HuH7 (**E**-**F**) and Hep3B (**G**-**H**) cells were incubated with XAV939 (**E** and **G**) or IWR-1 (**F** and **H**) at the indicated concentrations for 24 h. IFN-γ 25 ng/mL was then added for another 24 h before we harvested the cells

**Fig. 2:**
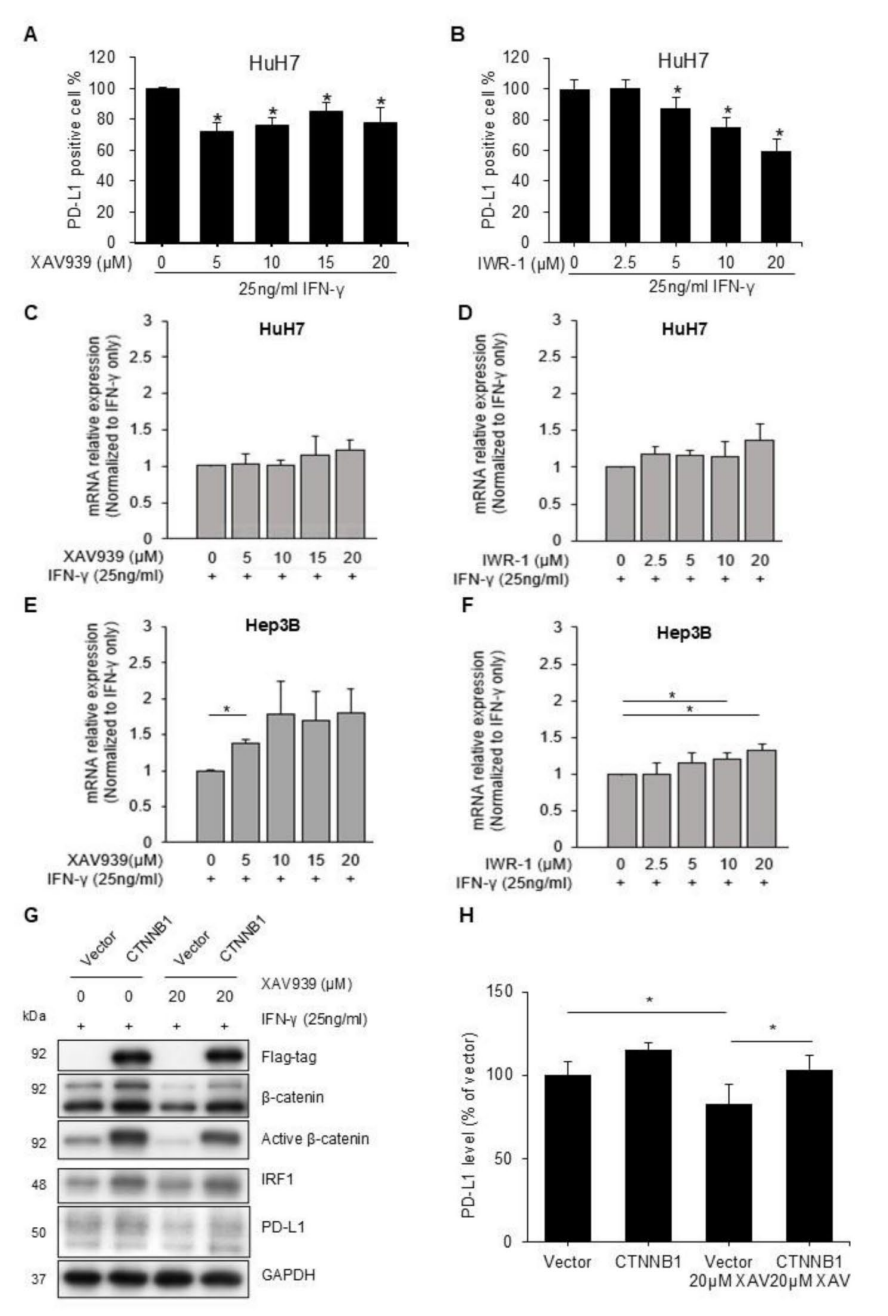
**(A-B)** Flow cytometry results revealing membranous PD-L1 expression after Wnt pathway inhibitor treatment. HuH7 cells were treated with XAV939 (**A**) or IWR-1 (**B**) at the indicated concentration for 24 h, before 25 ng/mL IFN-γ was added for another 24 h. The cells were subsequently harvested and stained with anti-PD-L1 antibodies or isotype controls (^*^p < 0.05). **(C-F)** Expression of *CD274* mRNA after Wnt pathway inhibitor treatment. HuH7 (**C**-**D**) and Hep3B (**E**-**F**) cells were treated with XAV939 (**C** and **E**) or IWR-1 (**D** and **F**) at the indicated concentration for 24 h, before 25 ng/mL IFN-γ was added for another 24 h. The cells were subsequently harvested. *CD274* mRNA expression was analyzed using RT-qPCR with GAPDH as the internal control. Expression is reported as fold changes relative to cells treated with the vehicle (^*^p < 0.05). **(G-H)** Western blotting demonstrating the influence of the Wnt pathway inhibitor XAV939 on PD-L1 expression in cells overexpressing beta-catenin. Empty vectors or vectors overexpressing *CTNNB1* (coding gene of beta-catenin) were added to the HuH7 cells after seeding. After 48 h, we treated the cells with XAV939 at the indicated concentration for 24 h before IFN-γ 25 ng/mL was added. The cells were harvested after 24 h. PD-L1 protein expression was analyzed with GAPDH as the internal control. Expression is reported as fold changes relative to cells treated with the empty vector and vehicle only (^*^p < 0.05)

Tankyrase inhibition stabilizes Axin and antagonizes Wnt signaling [21]. Using the reporter assay, we confirmed that tankyrase inhibitors, XAV939 and IWR-1, reduced Wnt pathway activity in human HCC cell lines HuH7 (Figs. 1A-B) and Hep3B (Figs. 1C-D). Although HuH7 and Hep3B exhibited low PD-L1 expression as reported [22], IFN-γ increased this expression (Figs. 1E-H). In HuH7 cells, XAV939 and IWR-1 both reduced IFN-γ induced PD-L1 expression in a dose-dependent manner, concurrently decreasing levels of active beta-catenin (unphosphorylated beta-catenin) (Figs. 1E-F). Similar results were observed in Hep3B cells (Figs. 1G-H). The downstream signal of IFN-γ, IRF-1, was also reduced by these Wnt pathway inhibitors. Flow cytometry confirmed that membranous PD-L1 expression decreased following treatment with either XAV939 or IWR-1 (Figs. 2A-B).

Despite the decrease in PD-L1 protein expression, XAV939 and IWR-1 did not reduce the mRNA levels of *CD274* (the coding gene for PD-L1) in HuH7 (Figs. 2C-D) and Hep3B (Figs. 2E-F) cells. This suggests that the reduction in PD-L1 expression could not be attributed to changes in IRF-1, which is known to influence mRNA expression of PD-L1.

Overexpressing beta-catenin, the key signaling molecule of the Wnt pathway, in HuH7 cells could reverse the reduction in PD-L1 expression caused by XAV939 (Figs. 2G-H) and IWR-1 (Figure S1), confirming that the observed effects were indeed due to Wnt pathway inhibition. Among 120 HCC tissue samples from patients, we found that the Wnt pathway activation, evaluated as nuclear translocation of beta-catenin, was associated with the higher percentage of PD-L1 positive cells among tumor cells (*p* = 0.05, Figure S2A) and all cells (*p* = 0.025, Figure S2B) in the tissue.

### Wnt pathway inhibition with *CTNNB1* knockdown yielded similar results

**Fig. 3:**
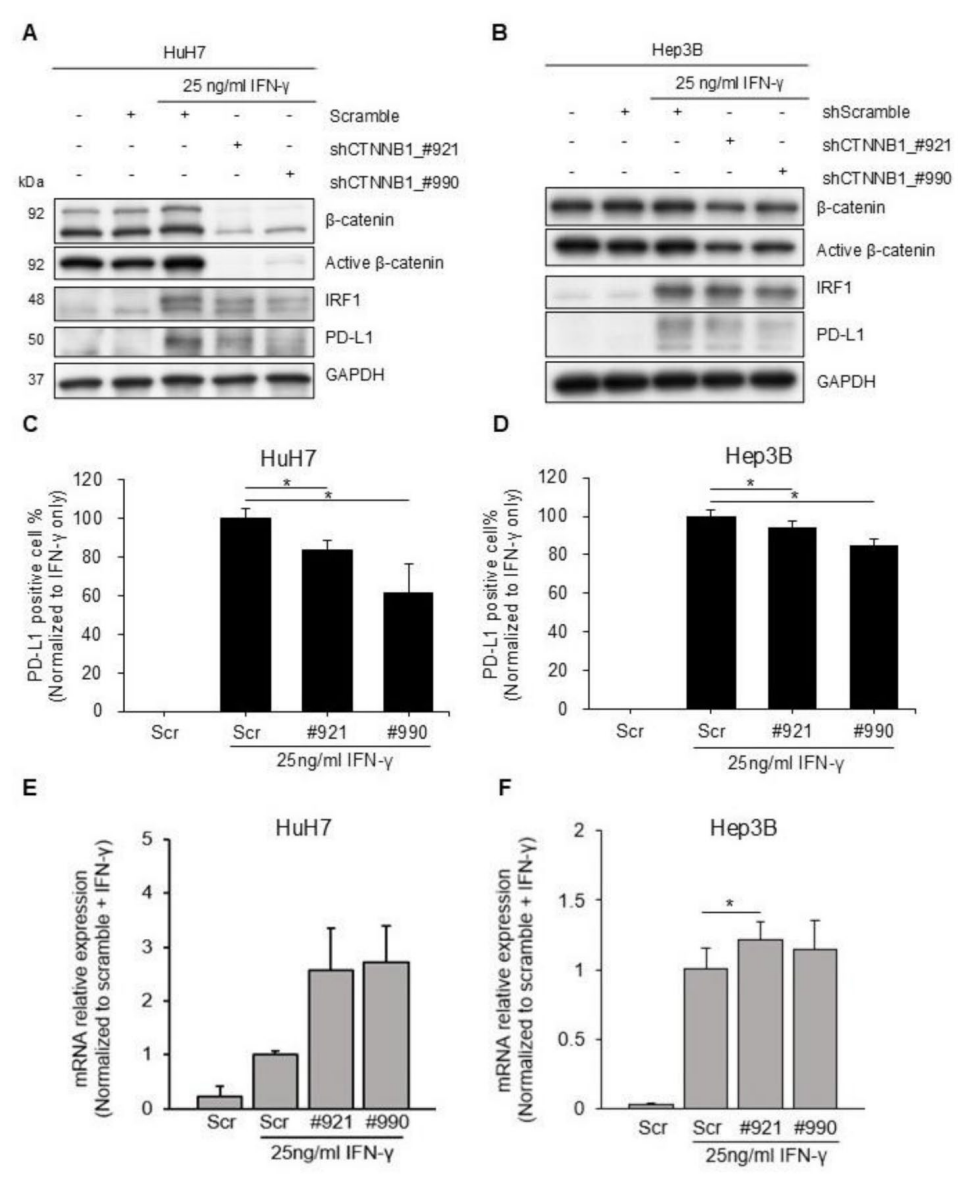
*CTNNB1* knockdown experiments. Approximately 16 h after seeding, *CTNNB1*-specific shRNAs or scramble shRNAs were added to HuH7 (**A**, **C**, **E**) and Hep3B (**B**, **D**, **F**) cells. After 48 h, IFN-γ 25 ng/mL was added. The cells were harvested after 24 h. **(A-B)** Western blotting demonstrating the influence of CTNNB1 knockdown on PD-L1 expression. **(C-D)** The harvested cells were stained with anti-PD-L1 antibodies or isotype controls. Flow cytometry was used to examine membranous PD-L1 expression. Expression is reported as fold changes relative to cells treated with scramble shRNAs (^*^p < 0.05). **(E-F)***CD274* mRNA expression was analyzed using RT-qPCR with GAPDH as the internal control. Expression is reported as fold changes relative to cells treated with the shRNAs (^*^p < 0.05)

We used shRNAs to knock down *CTNNB1* expression to confirm the findings with Wnt pathway inhibitors. *CTNNB1* knockdown reduced the IFN-γ induced PD-L1 expression in both HuH7 (Fig. 3A) and Hep3B (Fig. 3B) cells. Flow cytometry confirmed that membranous PD-L1 expression was also reduced following *CTNNB1* knockdown in HuH7 (Fig. 3C) and Hep3B (Fig. 3D) cells. However, similar to results using Wnt pathway inhibitors, *CD274* mRNA expression was not reduced by *CTNNB1* knockdown in HuH7 (Fig. 3E) and Hep3B (Fig. 3F) cells.

### Wnt pathway inhibition reduced PD-L1 translation through 4E-BP1

We examined multiple time points after Wnt pathway inhibition in HuH7 cells and observed a reduction in PD-L1 protein expression levels as early as 4 h after treatment (Figure S3A), while *CD274* mRNA expression remained stable (Figure S3B).

To determine if the reduction in PD-L1 protein level could be attributed to increased degradation, we used cycloheximide to halt translation. After cycloheximide treatment, PD-L1 protein expression levels were similar in cells treated with only vehicle and those treated with Wnt pathway inhibitors like XAV939 (Figures S4A and 4 A) and IWR-1 (Figures S4B and 4B). PD-L1 levels were also comparable between cells with and without *CTNNB1* knockdown after cycloheximide treatment (Figures S4C and 4 C), suggesting that the reduction in PD-L1 expression was due to decreased translation rather than increased degradation.

**Fig. 4:**
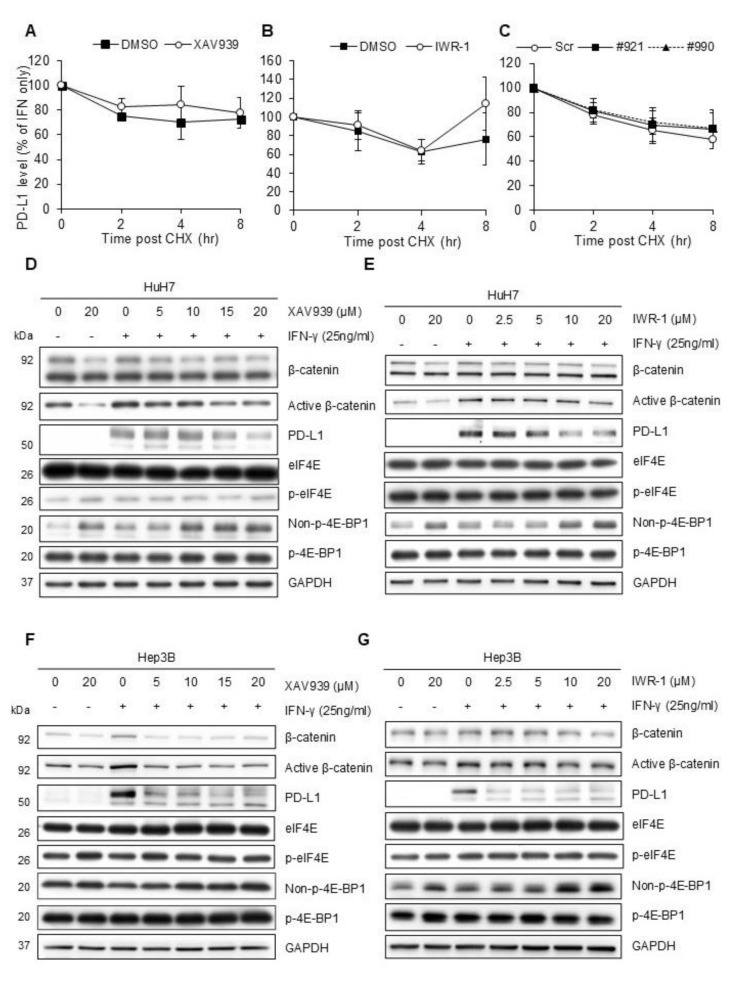
**(A-C)** Cycloheximide chase assays. Following Wnt pathway inhibitor XAV939 (**A**) and IWR-1 (**B**) treatment for 24 h or shRNA knockdown (**C**) for 48 h, HuH7 cells were stimulated with IFN-γ for 24 h. We then treated the cells with 20 µg/ml cycloheximide (CHX) for 0, 2, 4, and 8 h prior to cell harvest. Western blotting was utilized to detect the protein amount. PD-L1 level is reported as fold changes relative to cells treated with vehicle only (**A**-**B**) or scramble shRNAs (**C**) and without CHX. **(D-G)** Western blotting demonstrating changes of eIF-4E and 4E-BP1 levels along with PD-L1 expression after Wnt pathway inhibitor treatment. HuH7 (**D**-**E**) and Hep3B (**F**-**G**) cells were treated with XAV939 (**D** and **F**) or IWR-1 (**E** and **G**) for 24 h before 25 ng/mL IFN-γ was added. The cells were harvested after another 24 h

Literature on translation factors revealed that eukaryotic initiation factor 4E (eIF-4E) and its binding protein 4E-BP1 are associated with the Wnt pathway [23–26]. Unphosphorylated 4E-BP1 can bind to eIF-4E and inhibit its function in translation initiation. Following Wnt pathway inhibitor treatment, although the expression or phosphorylation of eIF-4E remained unchanged, unphosphorylated 4E-BP1 levels increased dose-dependently both in HuH7 (Figs. 4D-E) and Hep3B (Figs. 4F-G) cells. These results indicated that Wnt pathway inhibition reduced PD-L1 translation through increased levels of unphosphorylated 4E-BP1, thus blocking the translation initiation function of eIF-4E.

### In vivo model testing combined inhibition of Wnt and PD-L1 pathway

**Fig. 5:**
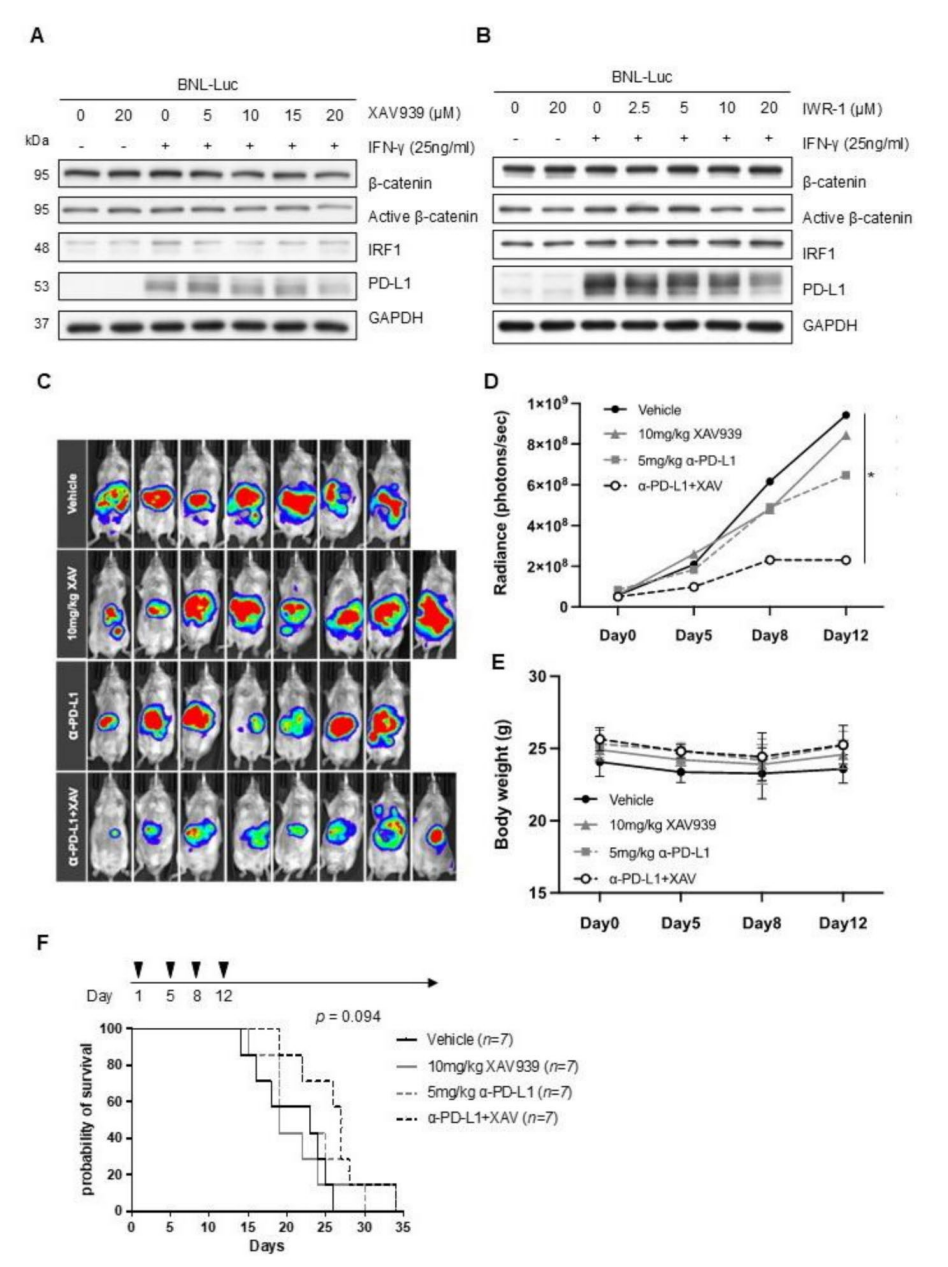
**(A-B)** Western blotting demonstrating mouse PD-L1 expression after Wnt pathway inhibitor treatment. XAV939 (**A**) or IWR-1 (**B**) at the indicated concentration were added to mouse BNL-Luc cells after seeding. After 24 h, mouse IFN-γ 25 ng/mL was added. The cells were harvested after another 24 h. **(C-F)** Orthotopic mouse HCC model. We implanted 3 × 10^5^ BNL-Luc cells in the subcapsular area of the liver in BALB/c mice. A week later, mice were randomized to receive vehicle (4% DMSO in corn oil and IgG control; *n* = 7), 10 mg/kg XAV939 (*n* = 8), 5 mg/kg anti-PD-L1 antibody (10 F.9G2, Bio X Cell, Lebanon, NH, USA; *n* = 7), or a combination of XAV939 and anti-PD-L1 antibody (*n* = 8). Treatment was given as intraperitoneal injections twice weekly for two weeks. Bioluminescence imaging was used to monitor the tumor sizes (**C**-**D**) (^*^p < 0.05). Mouse weights were also measured (**E**). In another separate experiment, the mice were not sacrificed unless they met the animal euthanasia criteria after 2 weeks of the indicated treatment. The Kaplan-Meier method was used to calculate survival from the day of treatment initiation. The log rank test was utilized to compare between groups (**F**)

Next, we tested whether the reduction in PD-L1 expression by Wnt pathway inhibition might enhance the treatment efficacy of anti-PD-L1 antibodies. Both XAV939 (Fig. 5A) and IWR-1 (Fig. 5B) were confirmed to reduce PD-L1 expression in the mouse HCC cell line BNL-Luc as well as in the human HCC cell lines HuH7 and Hep3B.

In the orthotopic mouse model, liver tumors were significantly smaller in mice treated with XAV939 in combination with an anti-PD-L1 antibody than in mice treated with vehicle only or either compound alone (Fig. 5C-D). The mice tolerated the treatment well, showing no significant weight differences among the treatment groups (Fig. 5E). Survival analysis revealed that mice treated with the combination of XAV939 and anti-PD-L1 antibody exhibited the longest survival (*p* = 0.019, Fig. 5F).

**Fig. 6:**
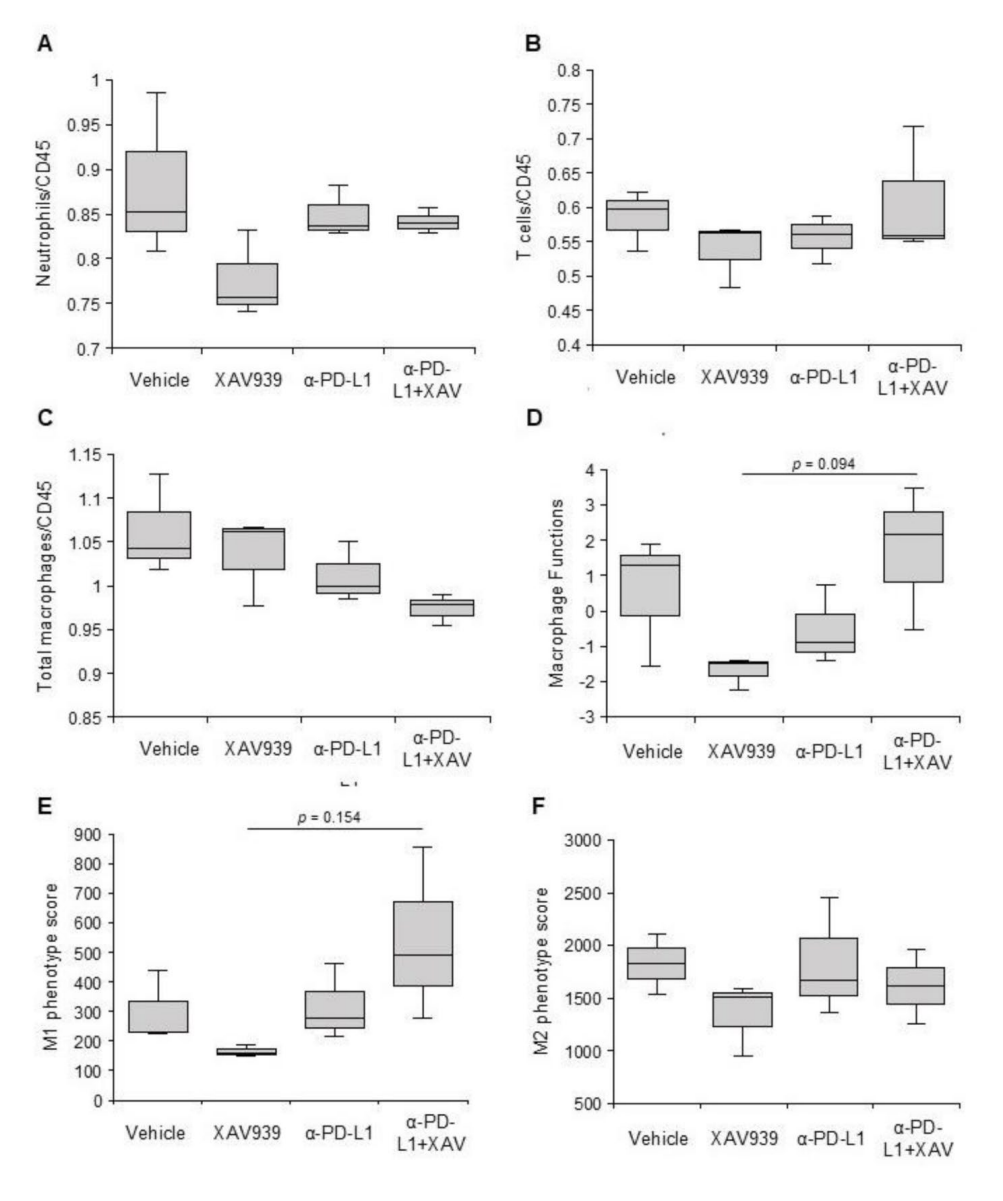
Immune-related gene expression profiling. After harvesting the tumors from the sacrificed mice, RNA was extracted and the PanCancer Immune Profiling Panel of NanoString nCounter^®^ analysis system was utilized to demonstrate the content of neutrophil (**A**), T cells (**B**), and total macrophages (**C**) in the tumors, as well as the macrophage function (**D**). We also analyzed the function of M1 and M2 macrophages (**E**-**F**), following the signatures previously published [27]. Boxes represent the 1st, 2nd and 3rd quartile, and the whisks represent the range

We examined immune-related gene expression in the harvested tumors using the PanCancer Immune Profiling Panel of the Nanostring nCounter^®^ analysis system. Tumors treated with XAV939 combined with an anti-PD-L1 antibody showed elevated function in multiple immune-related pathways, such as T-cell function and innate immunity (Figure S5), but the neutrophil, T-cell, and total macrophage contents remained stationary (Figs. 6A-C). Compared with tumors treated with single agents, tumors treated with both XAV939 and anti-PD-L1 antibody tended to have higher macrophage function (Fig. 6D). Exploring M1 and M2 macrophage functions separately using previously published M1 and M2 signatures [27], we found that the increased macrophage function in tumors treated with both XAV939 and anti-PD-L1 mainly came from M1 macrophages (Figs. 6E-F).

## Discussion

In this study, we demonstrated that Wnt pathway inhibition reduces IFN-γ-induced PD-L1 expression in HCC cells. Specifically, treatment with two Wnt pathway inhibitors, XAV939 and IWR-1, as well as shRNA targeting CTNNB1, uniformly reduced PD-L1 protein levels without affecting mRNA expression. In an orthotopic mouse model, we observed that Wnt pathway inhibition enhanced the treatment efficacy of anti-PD-L1 antibody, potentially through the activation of multiple immune-related pathways.

Combination therapy including PD-1 blockade is the current standard first-line therapy for advanced HCC, supported by multiple phase 3 clinical trials demonstrating survival benefits [1–6]. However, PD-L1 expression is typically low in HCC patient samples compared to other cancers, such as lung and urothelial cancers [28]. with a usual positivity cut point of 1% [3, 29, 30]. In addition, such positivity was not associated with overall survival benefits [3, 29]. Most HCC cell lines, including HuH7 and Hep3B used in our study, exhibit low PD-L1 expression in the absence of stimuli, mirroring clinical findings [21]. 

Upon interaction with the immune system, cancer cells can express immune checkpoints such as PD-L1 to evade antitumor immunity [18, 19, 31]. A previous study identified beta-catenin as a transcription factor for PD-L1 in HCC cells that consistently express PD-L1 [15]. In contrast, our study found that Wnt pathway inhibition did not reduce *CD274* transcription in cell lines with very low levels of PD-L1 expression, suggesting that consistent and inducible expressions of PD-L1 may be regulated through different mechanisms.

The impact of the Wnt pathway in HCC may extend beyond the tumor cells themselves. Activation of Wnt Pathway signaling has been linked to an immune desert landscape, immune exclusion, and resistance to immunotherapies in HCC [14, 32, 33]. In our orthotopic tumor model, the combination of Wnt pathway inhibitors and anti-PD-L1 antibody improved T-cell function, M1 macrophage activity, and innate immunity. This is consistent with reports that Wnt pathway activation promotes M2 macrophage polarization [34, 35], suggesting that Wnt pathway inhibition may enhance the efficacy of immunotherapy approaches beyond PD-1 blockade.

How tankyrase inhibition led to increased level of unphosphorylated 4E-BP1 was unclear. Although glycogen synthase kinase-3β (GSK-3β), a key component of the Wnt pathway, has been reported to phosphorylate 4E-BP1 [36, 37], the effect of tankyrase on GSK-3β outside the formation of the destruction complex and ubiquitination of beta-catenin was less known. Tankyrase function has also be linked to pathways other than Wnt, such as the Akt and YAP pathways [38]. Whether these pathways were also involved in the increased of unphosphorylated 4E-BP1 remained to be explored.

Our study is not without limitations. We investigated only two methods of Wnt pathway inhibition: tankyrase inhibition and *CTNNB1* knockdown. Although both approaches yielded consistent results, it remains to be seen whether other Wnt pathway inhibitors, such as porcupine inhibitors, would similarly affect PD-L1 expression. Furthermore, despite prolonged survival, mice treated with the combination of Wnt inhibitors and anti-PD-L1 antibody eventually succumbed to the disease, indicating that resistance to treatment still occurs. Future research should explore strategies to further delay or overcome resistance to immunotherapy. The influence of the Wnt pathway inhibition on the tumor microenvironment besides tumor cells might also contribute to the efficacy and warrants further study. The current immunoprofiling data originated from a limited number of samples and should be further validated and explored.

In conclusion, Wnt pathway inhibition reduced PD-L1 expression in HCC cells by inhibiting its translation and enhanced the efficacy of PD-1 blockade, highlighting a promising avenue for improving immunotherapy outcomes in HCC.

## Electronic supplementary material

Below is the link to the electronic supplementary material.

Supplementary Material 1

## Data Availability

No datasets were generated or analysed during the current study.
