# Supplementary material for: Downregulation of PD-L1 expression by Wnt pathway inhibition to enhance PD-1 blockade efficacy in hepatocellular carcinoma
Source: Biol Direct. 2025 Apr 10;20:49. doi: 10.1186/s13062-025-00645-8 (PMC11987266; doi:10.1186/s13062-025-00645-8)
Supplement: Supplementary file 1 — Supplementary Material 1 [file 13062_2025_645_MOESM1_ESM.pdf]

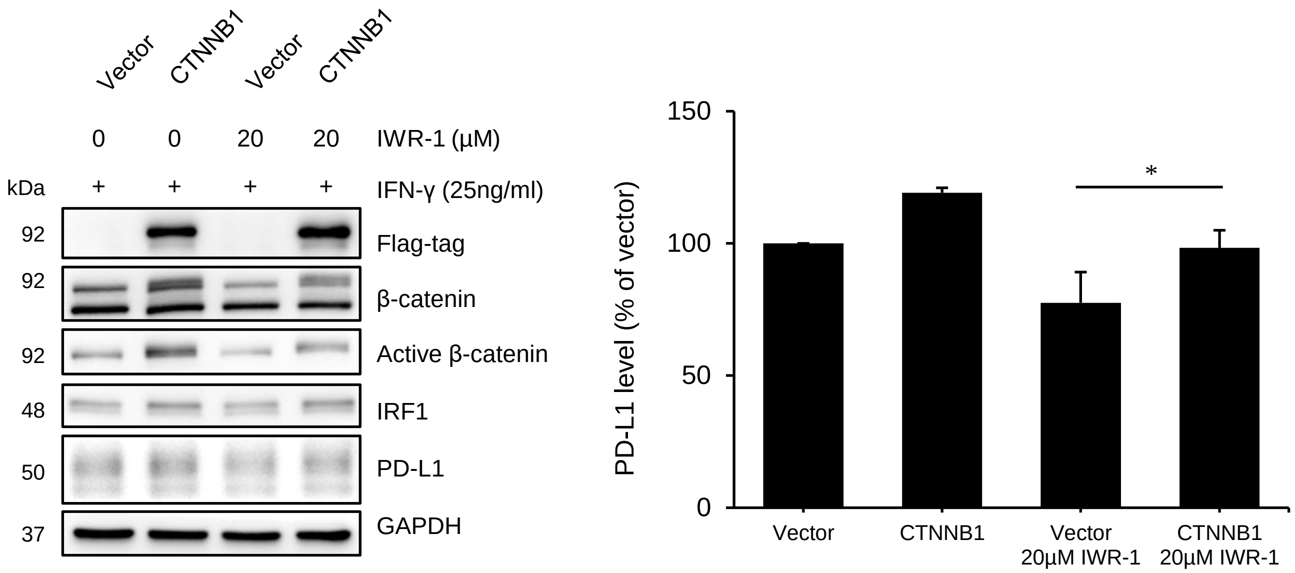

Figure S1. Western blotting demonstrating the influence of the Wnt pathway inhibitor IWR-1 on PD-L1 expression in cells overexpressing beta-catenin. Empty vectors or vectors overexpressing CTNNB1 (coding gene of beta-catenin) were added to the HuH7 cells after seeding. After 48 hours, we treated the cells with IWR-1 at the indicated concentration for 24 hours before IFN-γ 25 ng/mL was added. The cells were harvested after 24 hours. PD-L1 protein expression was analyzed with GAPDH as the internal control. Expression is reported as fold changes relative to cells treated with the empty vector and vehicle only (*$p < 0.05$).

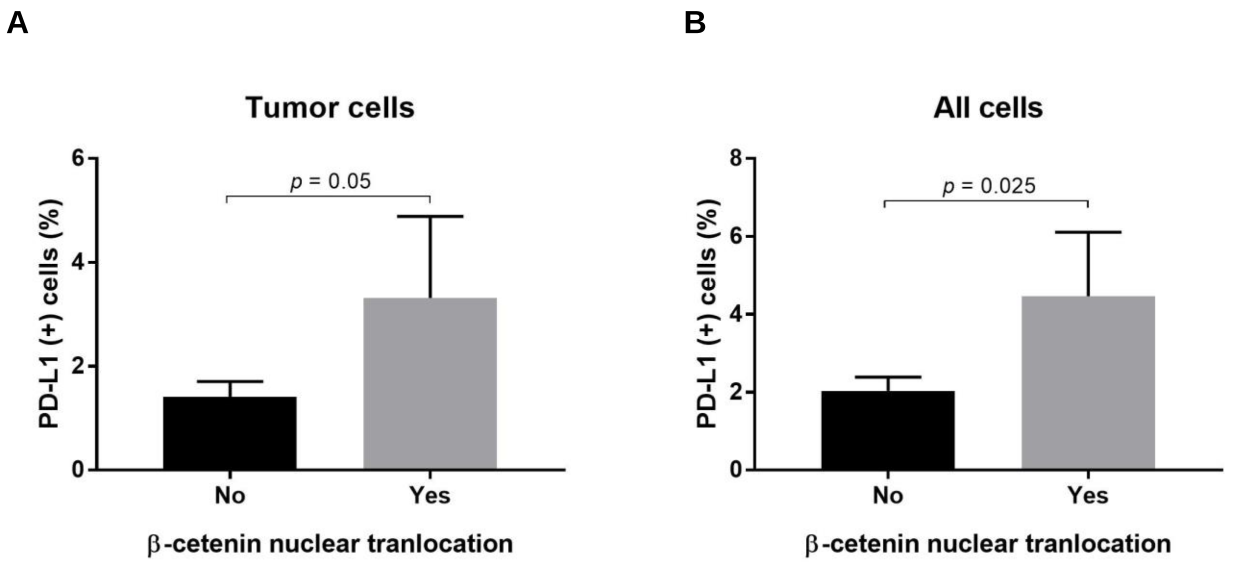

Figure S2. Immunohistochemical staining results of 120 HCC tissue samples from patients. The activity of the Wnt pathway was represented by the nuclear translocation of beta-catenin. PD-L1 expression was evaluated as the percentage of cells with positive staining.

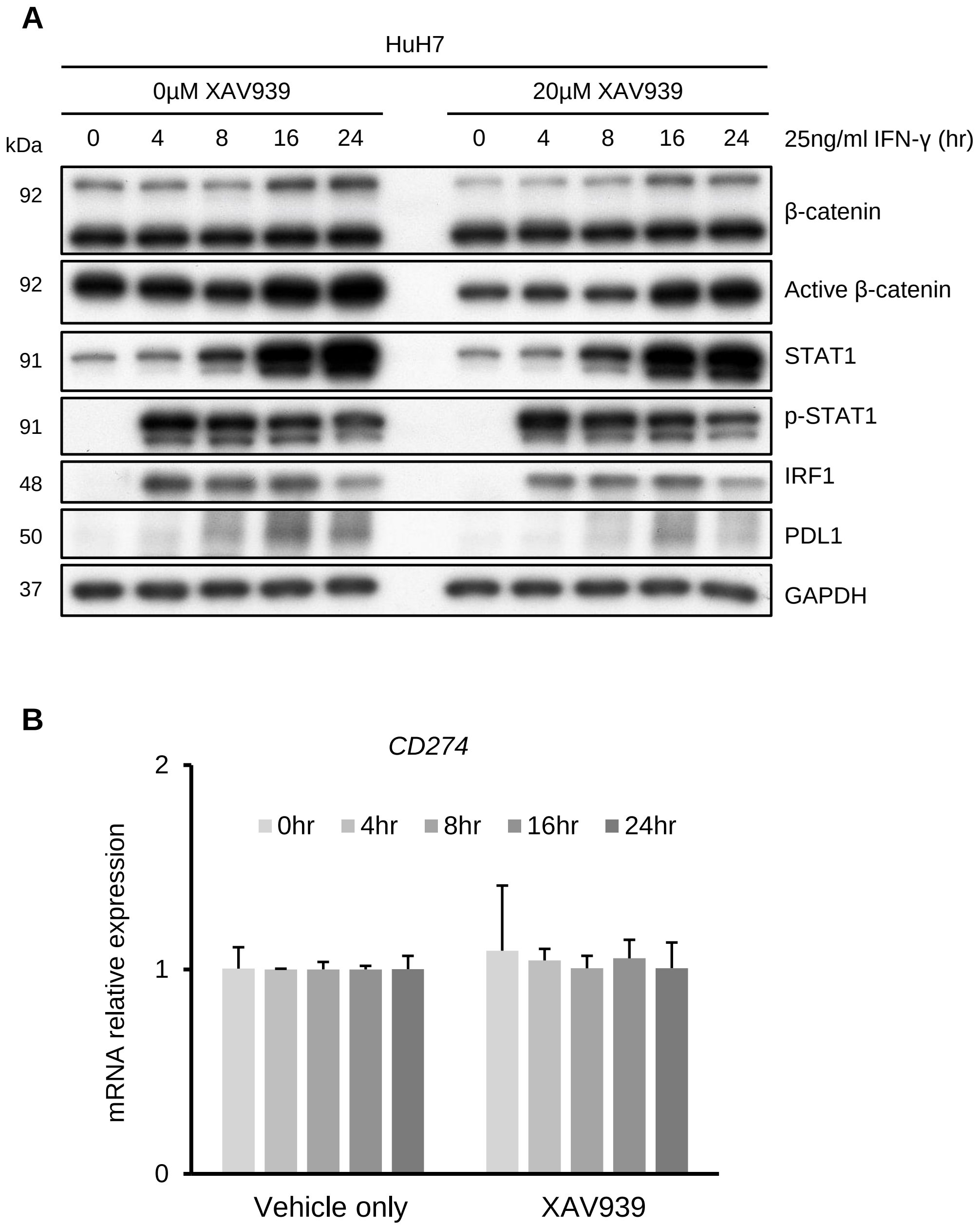

Figure S3. PD-L1 protein and mRNA expression in HCC cells after tankyrase inhibitor treatment. Approximately 16 hours after seeding, HuH7 cells were incubated with XAV939 20 μM for 24 hours. IFN-γ 25 ng/mL was then added for the indicated time before we harvested the cells. Western blotting (A) was utilized to demonstrate protein expression, and RT-PCR (B) was utilized for mRNA expression. CD274 mRNA expression was analyzed with GAPDH as the internal control. Expression is reported as fold changes relative to cells that were not treated with IFN-γ.

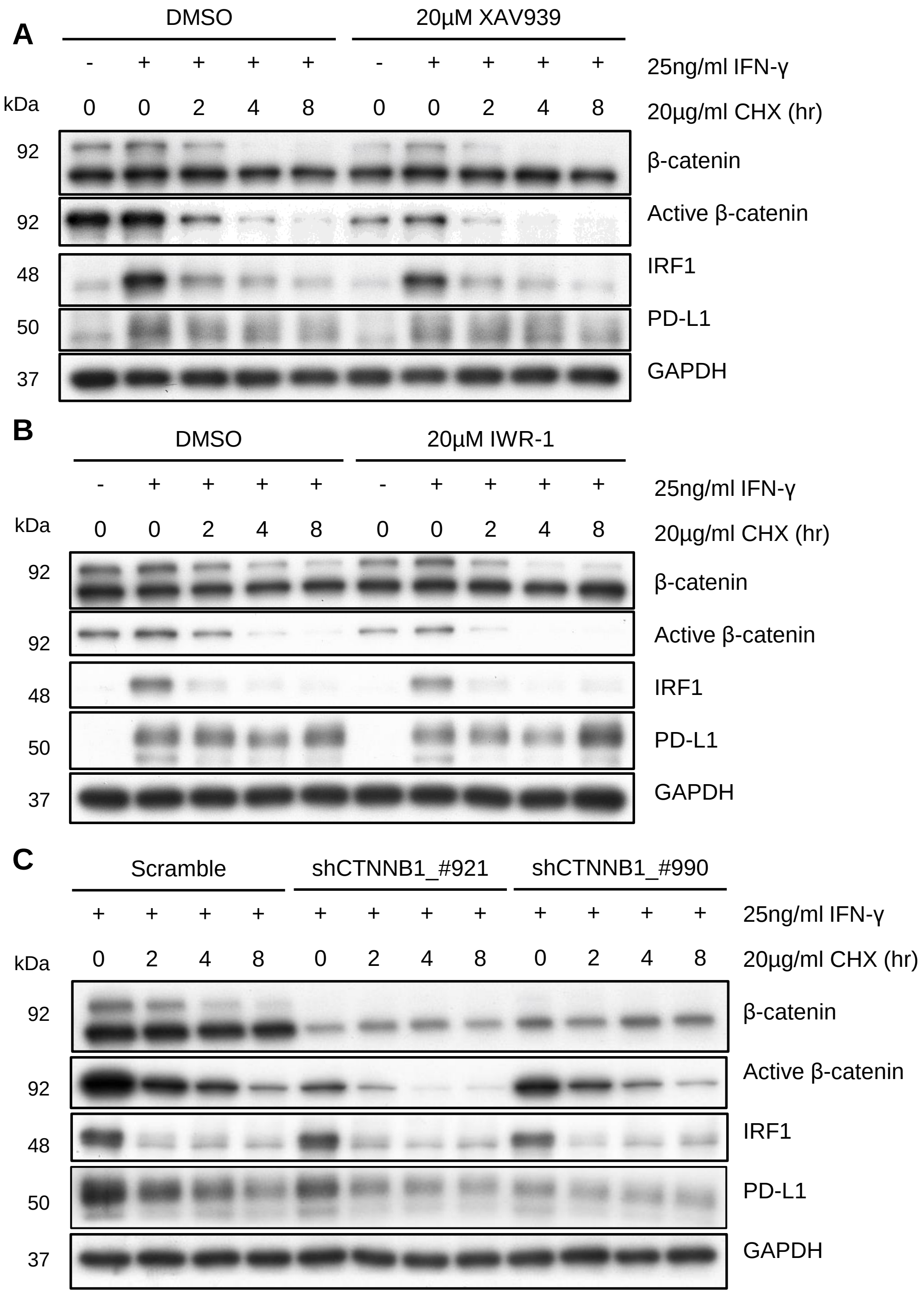

Figure S4. Cycloheximide (CHX) chase assays. Following tankyrase inhibitor XAV939 (A) and IWR-1 (B) treatment for 24 hours or shRNA knockdown (C) for 48 hours, HuH7 cells were stimulated with IFN-γ for 24 hours. We then treated the cells with 20 μg/ml cycloheximide for 0, 2, 4, and 8 hours prior to cell harvest. Western blotting demonstrated PD-L1 expression after treatment.

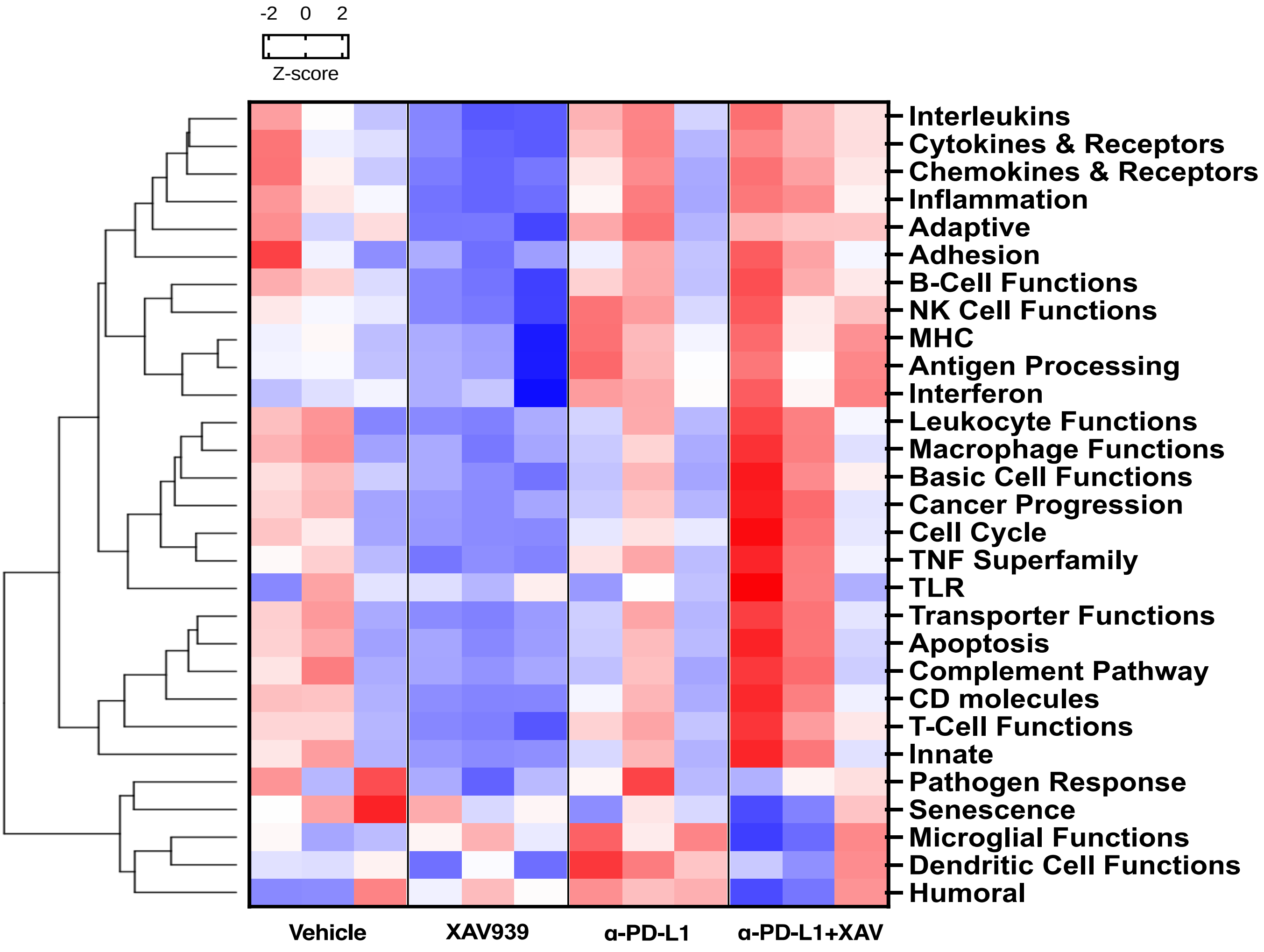

Figure S5. Immune-related pathway analysis in the orthotopic HCC model with BNL-Luc cells in BALB/c mice. Mice were randomized to receive vehicle only, 10 mg/kg XAV939, 5 mg/kg anti-PD-L1 antibody, or a combination of XAV939 and anti-PD-L1 antibody (all n = 3) twice weekly for two weeks. We then extracted RNA of the harvested tumors and utilized the PanCancer Immune Profiling Panel of Nanostring nCounter® analysis system for analysis.
